# 30-day hospital admission among older adults initially managed at home by a mobile emergency unit: a retrospective cohort study

**DOI:** 10.1186/s12873-026-01536-5

**Published:** 2026-03-11

**Authors:** Masoud Moradi, Anita Maués Østergaard Pedersen, Katrine Jaquet Mavraganis, Mette Rahbek Kristensen, Johanne Overgaard Wessels, Sina Bayatshahbazi, Peter Biesenbach

**Affiliations:** 1 Department of Emergency Medicine, University Hospital of Southern Denmark Esbjerg, Esbjerg, Denmark; 2 Research Unit of Emergency Medicine, University Hospital of Southern Denmark Esbjerg, Esbjerg, Denmark; 3 Department of Medicine, Basildon University Hospital, Basildon, Essex, UK

**Keywords:** Prehospital care, Home-based assessment, Emergency medicine, Older adults, Hospital admission, Mobile emergency unit

## Abstract

**Background:**

In recent years, Denmark has introduced mobile emergency unit (MEU) to provide patients with home-based evaluation and treatment by emergency medicine physicians. The aim is to avoid unnecessary hospital admissions and to reduce overcrowding in emergency departments. However, it is unknown which demographic, clinical, and paraclinical characteristics of patients at the index MEU assessment are related to subsequent hospital admission. Therefore, we aimed to describe these baseline characteristics and to examine their association with 30-day hospital admission.

**Methods:**

In this retrospective, single-centre cohort study at Esbjerg Hospital (Region of Southern Denmark), we screened 1656 MEU contacts (from 1 January to 31 December 2024) and included adults aged ≥ 65 years, who were not directly admitted/conveyed to hospital at the index visit (i.e. initially managed at home). These patients were potential candidates for hospital admission, and the emergency physician made an on-scene decision regarding admission. Data were analysed using multivariable logistic regression.

**Results:**

We included 357 MEU contacts, with a median (interquartile range) age of 83.5 (77.6–89.2) years. 140 (39.2%) of these contacts were admitted to hospital within 30 days. A higher proportion of the admitted patients had a pre-existing do-not-attempt-resuscitation (DNAR) order compared with the non-admitted patients (85.0% vs 66.4%; *p* < 0.001) and lived at home (57.8% vs. 47.4%; *p* = 0.055). Chronic pulmonary disease was more common among the admitted patients (31.4% vs 19.3%; *p* = 0.009), whereas dementia was less frequent (18.6% vs 28.1%; *p* = 0.042). Both a pre-existing DNAR order (odds ratio [OR] 3.83, 95% confidence interval [CI] 2.05–7.16) and home (vs nursing home) residence (OR 1.76, 95% CI 1.03–2.98) were significantly associated with hospital admission in the adjusted model.

**Conclusions:**

Among older adults assessed at home by MEU physicians, a pre-existing DNAR order and home (vs nursing home) residence were independently associated with 30-day hospital admission. These findings may inform triage and follow-up planning. However, prospective studies are required to establish causal links.

**Supplementary Information:**

The online version contains supplementary material available at 10.1186/s12873-026-01536-5.

## Background

The global population is ageing rapidly: by 2050, the number of adults aged 60 years and above is projected to double [1]. This situation has led to major challenges for healthcare systems, particularly emergency departments (EDs) [2–4]. Older adults are especially vulnerable to acute illness, multimorbidity, and polypharmacy, which increase their risk of adverse hospital-related outcomes, including delirium, nosocomial infections, functional decline, and mortality [5–9]. Denmark has aimed to address this situation by implementing mobile emergency unit (MEU). As part of this prehospital initiative, an emergency medicine (EM) physician and nurse conduct clinical assessments and initiate treatment in the patient’s residence. This approach aims to prevent unnecessary hospital admissions and reduce the associated risks with admission [10–14]. Emerging data suggest that evaluation by MEU allows a substantial proportion of older adults to be managed safely at home following the initial assessment and intervention [14].

There has been extensive work to predict hospital admission, readmission, or length of stay. Researchers have developed prognostic models based on ED triage data, prehospital records, and/or post-discharge information. Many of these models depend on demographic characteristics, comorbidity indices, and vital signs [15–20]. However, there are some important issues with these models. First, there has been limited work on developing models specifically tailored to older adults, a particularly relevant issue given population ageing. A notable example is an ED-based tool proposed by Abugroun et al. [21] that is tailored to older adults. It integrates both clinical and functional measures to support decisions as to whether patients should be admitted to hospital. Second, it would also be useful to integrate findings from point-of-care ultrasound (POCUS) and point-of-care testing (POCT) into these models. Currently, POCUS is generally used to assess specific conditions (e.g., dyspnoea), and its ability to stratify older adults based on hospital admission risk has not been widely evaluated [22–26]. Likewise, POCT offers rapid diagnostics but has seldom been combined with other prehospital data to create a comprehensive risk model.

Identifying higher-risk patients at the time of a home visit is important to ensure timely conveyance and hospital admission when needed, while lower-risk patients may be managed safely at home with appropriate follow-up. In the MEU setting, clinicians must often make rapid disposition decisions during a single home assessment, typically with limited opportunities for prolonged observation and diagnostics mainly restricted to bedside tools such as POCT and POCUS. Better risk stratification at the point of care could therefore improve patient safety and resource allocation and help refine MEU escalation and follow-up pathways. However, it remains unclear which baseline demographic, clinical, and paraclinical features recorded during EM physician–led home visits are associated with subsequent admission. Therefore, we aimed to compare baseline characteristics between older adults with and without 30-day hospital admission after an MEU home-based assessment and to estimate adjusted associations using multivariable logistic regression

## Methods

### Study design and setting

We conducted a retrospective, single-centre cohort study and reported the results in accordance with Strengthening the Reporting of Observational Studies in Epidemiology (STROBE) guidance [27]. We screened all MEU contacts between 1 January 2024 and 31 December 2024 in the catchment area of Esbjerg Hospital, Region of Southern Denmark. The MEU covers approximately 3400 km^2^ and serves a population of about 240,000.

### Description of the MEU service

MEU-based care, operated by the Esbjerg Hospital ED, forms an integral part of the regional prehospital care system. It is staffed by an EM physician and a nurse and is available daily from 08:00 to 18:00. There are two ways to activate the MEU: referral from a general practitioner, on-call physician, or nursing home to the ED admission coordinator; or direct activation by the emergency medical dispatch centre if a 1–1-2 call call is assessed as suitable for MEU response according to regional dispatch protocols. In brief, MEU activation is intended for patients who are clinically stable and where there is no suspicion of an immediately life-threatening condition requiring urgent ambulance transport or advanced prehospital resuscitation, and where assessment and initial treatment in the patient’s residence is deemed appropriate. Upon arrival, the MEU conducts an on-site emergency assessment. They may initiate treatment (e.g., intravenous antibiotics or fluids) and retain medical responsibility for up to 96 hours after their visit, with follow-up visits as clinically indicated. The team works in collaboration with municipal acute care nurses, who continue prescribed medication or monitoring, and escalate to the ED if the patient deteriorates.

### Participants

**Fig. 1:**
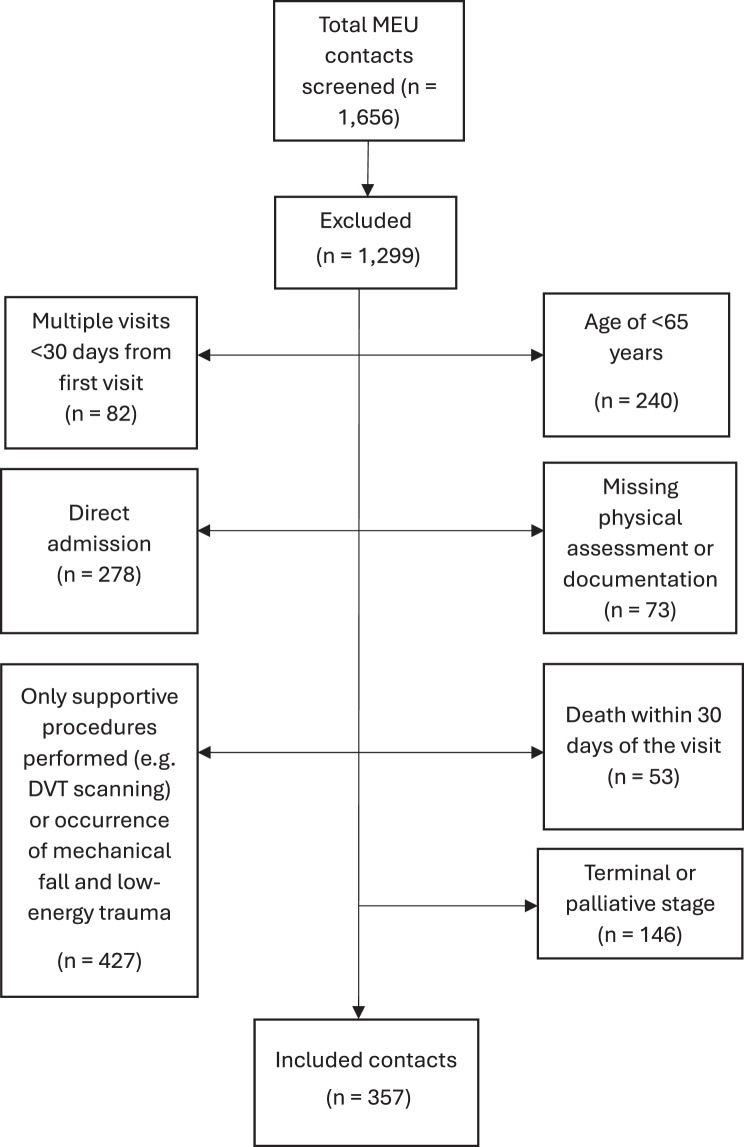
Study cohort selection and exclusions from MEU contacts. Flow diagram showing screening of 1656 mobile emergency unit (MEU) contacts in 2024 and reasons for exclusion, resulting in 357 index contacts analyzed for the primary outcome. DVT, deep vein Thrombosis; MEU, mobile emergency unit

We screened all 1656 MEU contacts during the study period and patients were excluded based on the following criteria (Fig. 1):

- Age < 65 years;
- When patients were not considered candidates for hospital admission due to pre-existing comorbidities, a severely reduced functional status, or limited life expectancy, or were receiving palliative care for terminal illness;
- Incomplete or missing MEU documentation in the electronic health record (EHR);
- 30-day mortality and competing events: Thirty-day mortality (including death during the index contact or within 30 days thereafter) was ascertained for all patients. For the primary endpoint of 30-day hospital admission, patients who died within 30 days without any hospital admission were considered a competing event for admission and were not included in the admission-risk analysis (*n* = 53). Patients who were admitted within 30 days and subsequently died were retained in the admission analysis (*n* = 31).
- Contact limited to a telephone consultation (no physical assessment);
- Only technical or supportive procedures performed without clinical assessment or decision-making (e.g., peripheral venous catheter, urinary catheter, nasogastric tube, blood sampling, wound care, or wound assessment);
- Direct admission/conveyance to hospital at the index MEU visit (i.e., patients not initially managed at home after the first MEU assessment)
- Assessment for low-energy trauma (e.g., head or extremity injury) where the team concluded the injury resulted from a mechanical fall and no further on-scene diagnostics were performed. In our setting, MEU-assessed trauma cases largely represent very low-risk minor injuries (as moderate/high-risk trauma is typically conveyed directly to hospital). Documentation of vital parameters and on-scene tests (POCT/POCUS) is frequently limited in this subgroup; including these visits would therefore substantially increase missing data and introduce a risk of information bias;
- Referral solely for deep vein thrombosis (DVT) scanning without other acute complaints or interventions;

When a patient had multiple MEU visits, we only included the first visit. If a subsequent visit occurred more than 30 days later, we considered it a new case.

### Data sources and variables

We extracted data from EHRs and, when needed, cross-checked their completeness and accuracy against the emergency medical dispatch centre records (e.g., visit times and patient identifiers). We collected the following variables:

- Demographics, including age, sex, place of residence (home or nursing home), date of death (if applicable), smoking status, and alcohol use disorder;
- The do-not-attempt-resuscitation (DNAR) status based on documented decisions prior to the MEU visit;
- Pre-existing comorbidities were extracted from the electronic health record based on documented diagnoses and included hypertension, diabetes mellitus, cancer (none/prior/metastatic), chronic pulmonary disease, and dementia. Comorbidity burden was defined as the number of these predefined comorbidity domains and categorised as 0, 1–2, or ≥3. We did not calculate a weighted comorbidity index (e.g., Charlson Comorbidity Index), as our aim was a pragmatic measure based on routinely available information during prehospital decision-making;
- Data from the index visit, including the reason for referral and use/findings of POCT and POCUS (obtained from clinical notes when available), were extracted; findings were frequently documented qualitatively in the notes (e.g., normal/abnormal based on the local reference range). POCT comprised C-reactive protein (CRP) and venous blood gas (VBG) analyses performed on scene as clinically indicated.

Note that when the chief complaints are cardiological, the patients are generally routed directly to hospital. Thus, cardiological patients are under-represented in our sample. The case mix primarily reflects respiratory, urinary, neurological, and other presentations.

### Outcome

The primary outcome was 30-day hospital admission following initial home management. We defined this as the first hospital admission within 30 days among patients who were initially assessed by the MEU and judged suitable to remain at home. We determined the outcome from the EHRs and administrative records.

### Data categorisation

Because many of the available measurements were either charted qualitatively or not recorded, we coded all physiological parameters (systolic blood pressure, oxygen saturation, heart rate, temperature, and the Glasgow Coma Scale [GCS] score) and tests (POCT and POCUS) as three-level categorical variables: normal, abnormal, or not performed. We defined the abnormal values by using prespecified cut-offs: systolic blood pressure of <100 mmHg, oxygen saturation of <92% (or < 88% in patients with documented chronic pulmonary disease), heart rate of < 50 or >100 beats per minute (bpm), temperature of ≥38 °C, and a GCS score of <15. We excluded the respiratory rate because it was missing for a large number of cases. We retained the not recorded/not performed category because omission of POCT may reflect clinical judgement and/or documentation practices and may therefore carry prognostic information. If no POCT result was recorded in the MEU documentation, POCT was coded as not recorded/not performed. In this retrospective dataset, absence of a recorded POCT result was treated as not performed; however, this category may also reflect incomplete documentation and should therefore be interpreted with caution.

### Statistical analysis

We used IBM SPSS Statistics, version 26 (IBM Corp., Armonk, NY, USA) for all data analysis. The categorical variables are presented as counts and percentage, while the continuous variables are summarised as the mean or median and interquartile range (IQR), as appropriate. We used the Mann–Whitney U test and the χ^2^ test to compare between groups. We also performed univariable logistic regression for descriptive purposes. Candidate predictors were prespecified a priori based on clinical relevance and the literature and were included in a multivariable binary logistic regression model (i.e., we did not select them solely based on the *p*-values from univariable regression). *p*-values < 0.05 were considered statistically significant.

### Ethical considerations

This study was approved as a quality-improvement project by the Esbjerg Hospital administration and registered in the internal registry of the Region of Southern Denmark. In line with Danish legislation and guidance from the National Committee on Health Research Ethics, this retrospective, non-interventional study did not require formal approval by an ethics committee. The study complied with the Declaration of Helsinki and the General Data Protection Regulation.

## Results

In total, we included 357 contacts that met the eligibility criteria. Table 1 shows the baseline characteristics of the patients. The median (IQR) age of all patients was 83.5 (77.6–89.2) years. Age did not differ significantly between those admitted to hospital within 30 days of the index visit (81.8 (IQR = 76.2–89.3) years) and those not admitted (84.2 (IQR = 77.9–88.9) years; *p* = 0.334). Of the included contacts, 162 (45.4%) were men and 195 (54.6%) were women. The gender distribution did not differ significantly between those who were admitted to hospital within 30 days of the index visit and those who were not (*p* = 0.908). Among patients admitted within 30 days (*n* = 140), the median time from the index MEU contact to first hospital admission was 4 days (IQR 1–10); the distribution is shown in Additional file 1.

**Table 1:** Baseline characteristics of the study population stratified by 30-day hospital admission status

	Not admitted (n = 217)	Admitted (n = 140)	Total(n = 357)	p-value
Age, years	84.2 (77.9-88.9)	81.8 (76.2-89.3)	83.5 (77.6-89.2)	0.334
Sex				
Male	99 (45.6)	63 (45)	162(45.3)	0.908
Female	118 (54.4)	77 (55)	195(54.7)	
Place of residence				
Home	103 (47.4)	81 (57.8)	184(51.5)	0.055
Nursing home	114 (53)	59 (42.2)	173(48.5)	
Pre-existing DNAR				
No	73 (33.6)	21 (15)	94(26.3)	<0.001
Yes	144 (66.4)	119 (85)	263(73.7)	
Hypertension				
Yes	110 (50.7)	77 (55)	187(52.3)	0.426
No	107 (49.3)	63 (45)	170(47.6)	
Diabetes mellitus				
Yes	42 (19.3)	39 (27.9)	81(22.6)	0.061
No	175 (80.7)	101 (72.1)	276(77.4)	
Cancer				
No	176 (81.1)	108 (77.1)	284(79.6)	
Prior	34 (15.7)	24 (17.1)	58(16.2)	0.464
Metastatic	7 (3.2)	8 (5.8)	15(4.2)	
Chronic pulmonary disease				
Yes	42 (19.3)	44 (31.4)	86(24.1)	0.009
No	175 (80.7)	96 (68.6)	271(75.1)	
Dementia				
Yes	61 (28.1)	26 (18.6)	87(24.4)	0.042
No	156 (71.9)	114 (81.4)	270(75.6)	
Comorbidity burden				
0	45 (20.7)	23 (16.4)	68(19.1)	
1–2	155 (71.4)	97 (69.3)	252(70.5)	0.116
≥3	17 (7.9)	20 (14.3)	37(10.4)	
Smoking status				
Current smoker	18 (8.3)	24 (17.1)	42(11.8)	
Former smoker	62 (28.6)	50 (35.7)	112(31.4)	0.005
Never smoker	48 (22.1)	30 (21.5)	78(21.8)	
Unknown	89 (41)	36 (25.7)	125(35)	
Alcohol use disorder				
Yes	9 (4.1)	11 (7.9)	20(5.6)	0.137
No or unknown	208 (95.9)	129 (92.1)	337(94.4)	
Primary presentation complain				
Respiratory	85 (39.2)	56 (40)	141(39.5)	
Urinary	23 (10.6)	16 (11.4)	39(10.9)	0.977
Neurological	36 (16.6)	21 (15)	57(16)	
Other	73 (33.6)	47 (33.6)	120(33.6)	

Data are presented as median [IQR] or n (%). DNAR, do-not-attempt-resuscitation; IQR, interquartile range

### Univariable analysis

Univariable logistic regression (Table 2) revealed that the following variables led to higher odds of 30-day hospital admission: chronic pulmonary disease (odds ratio [OR] 1.91, 95% confidence interval [CI] 1.16–3.11; *p* = 0.010), a pre-existing DNAR order (OR 2.87, 95% CI 1.67–4.94; *p* < 0.001), and a comorbidity burden ≥ 3 versus 0 (OR 2.30, 95% CI 1.01–5.22; *p* = 0.046). Dementia was associated with lower odds of 30-day hospital admission (OR 0.58, 95% CI 0.34–0.98; *p* = 0.042) and an abnormal POCT finding with higher odds (OR 1.63, 95% CI 1.01–2.64; *p* = 0.045). Home (vs nursing-home) residence showed a trend for higher odds of 30-day hospital admission (OR 1.51, 95% CI 0.99–2.32; *p* = 0.055). The other variables were not significantly associated with 30-day admission (*p* > 0.05).

**Table 2:** Univariable logistic regression analysis for predictors of 30-day hospital admission

	OR	95% CI	p-value
Age (per year)	0.98	0.96–1.01	0.308
Sex			
Male (baseline)			
Female	1.02	0.66–1.57	0.908
Place of residence			
Nursing home (baseline)			
Home	1.51	0.99–2.32	0.055
Hypertension			
No (baseline)			
Yes	1.18	0.77–1.82	0.426
Diabetes mellitus			
No (baseline)			
Yes	1.61	0.97–2.65	0.062
Cancer			
No (baseline)			
Prior	1.15	0.64–2.04	0.633
Metastatic	1.86	0.65–5.28	0.242
Chronic pulmonary disease			
No (baseline)			
Yes	1.91	1.16–3.11	0.010
Dementia			
No (baseline)			
Yes	0.58	0.34–0.98	0.042
Comorbidity burden			
0 (baseline)			
1–2	1.22	0.69–2.15	0.481
≥3	2.31	1.01–5.22	0.046
Pre-existing DNAR			
No (baseline)			
Yes	2.87	1.67–4.94	<0.001
Smoking status			
Never (baseline)			
Unknown	0.64	0.35–1.17	0.154
Former	1.29	0.71–2.32	0.396
Current	2.13	0.99–4.57	0.051
Alcohol use disorder			
No (baseline)			
Active or former	1.97	0.79–4.88	0.143
Temperature			
Normal (baseline)			
Abnormal	1.08	0.56–2.11	0.803
Not recorded/not performed	0.85	0.51–1.43	0.555
Oxygen saturation			
Normal (baseline)			
Abnormal	1.18	0.63–2.24	0.594
Not recorded/not performed	1.05	0.61–1.81	0.838
Blood pressure			
Normal (baseline)			
Abnormal	2.05	0.85–4.92	0.106
Not recorded/not performed	1.36	0.77–2.38	0.283
GCS score			
Normal (baseline)			
Abnormal	1.21	0.74–­1.99	0.436
Not recorded/not performed	0.96	0.49–1.88	0.911
Heart rate			
Normal (baseline)			
Abnormal	1.06	0.49–2.28	0.879
Not recorded/not performed	1.48	0.86–2.56	0.153
POCT			
Normal (baseline)			
Abnormal	1.63	1.01–2.64	0.045
Not recorded/not performed	1.79	0.91–3.49	0.088
POCUS			
Normal (baseline)			
Abnormal	2.04	0.95–4.36	0.065
Not recorded/not performed	1.45	0.86–2.44	0.162

Odds ratios (OR) and 95% confidence intervals (CI) from univariable logistic regression models. CI, confidence interval; DNAR, do-not-attempt-resuscitation; OR, odds ratio; POCT, point-of-care testing; POCUS, point-of-care ultrasound

### Multivariable logistic regression

We fitted a multivariable logistic regression model using the following prespecified baseline predictors: age, sex, place of residence, pre-existing DNAR, the smoking status (unknown, former, or current vs never), alcohol use disorder, comorbidity burden, diabetes mellitus, chronic pulmonary disease, dementia, and the POCT category (abnormal or not performed vs normal). The model was statistically significant (χ^2^ [df = 15] = 50.65, *p* < 0.001), showed acceptable fit (Hosmer–Lemeshow χ^2^ [df = 8] = 8.89, *p* = 0.352), and demonstrated moderate explanatory power (Nagelkerke R^2^ = 0.181). Based on the model, two factors showed an independent association with 30-day admission: home (vs nursing-home) residence (OR 1.76, 95% CI 1.03–2.98, *p* = 0.037) and a pre-existing DNAR order (OR 3.83, 95% CI 2.05–7.16, *p* < 0.001). The other variables were not independently associated after adjustment (*p* > 0.05) (Table 3).

**Table 3:** Multivariable logistic regression identifying independent predictors of 30-day hospital admission

	OR	95% CI	p-value
Age	0.98	0.95–1.01	0.370
Female sex	1.19	0.73–1.95	0.470
Place of residence	1.76	1.03–2.98	0.037
Diabetes mellitus	1.51	0.73–3.06	0.262
Chronic pulmonary disease	1.09	0.55–2.14	0.793
Dementia	0.73	0.37–1.43	0.372
Comorbidity burden			
1–2	0.98	0.48–1.98	0.956
≥3	1.47	0.41–5.34	0.553
Pre-existing DNAR	3.83	2.05–7.16	<0.001
Smoking status			
Unknown	0.58	0.29–1.14	0.117
Former	0.91	0.45–1.81	0.789
Current	1.69	0.66–4.31	0.267
Alcohol use disorder	0.57	0.20–1.64	0.305
POCT			
Abnormal	1.66	0.98–2.82	0.059
Not recorded/not performed	1.92	0.93–3.96	0.076

OR, odds ratio; CI, confidence interval; DNAR, do-not-attempt-resuscitation; POCT, point-of-care testing. ORs are adjusted for all covariates shown. Reference categories were: male sex, nursing home residence, no diabetes mellitus, no chronic pulmonary disease, no dementia, comorbidity burden = 0, no pre-existing DNAR, never smoker, no alcohol use disorder, and normal POCT. For POCT, “not recorded/not performed” indicates absence of a recorded CRP and/or VBG result in the MEU documentation

## Discussion

In this retrospective cohort study, we identified variables associated with 30-day hospital admission among older adults assessed and initially managed at home by the MEU, and notably, we found that a pre-existing DNAR order and home (vs nursing home) residence were associated with increased odds of admission. POCT findings showed a trend towards higher odds of admission but did not reach conventional statistical significance after adjustment and should therefore be interpreted with caution, particularly given potential documentation-related missingness. These findings highlight the clinical and contextual factors that shape post-evaluation trajectories in this novel prehospital care setting.

There have been equivocal findings regarding the association of the DNAR status with hospitalisation and mortality. Differently from our study, Mehta et al. [28] reported that an early DNAR order in patients hospitalised with pneumonia was associated with a reduced risk of unplanned 30-day readmission. However, in the surgical setting, a newly established DNAR order during hospitalisation was linked to substantially higher postoperative mortality and increased rates of serious complications, including pneumonia, stroke, and myocardial infarction [29]. Similarly, Sheehan et al. [30] demonstrated that patients who had a DNAR order upon hospital admission had significantly higher in-hospital mortality and lower utilisation of intensive interventions compared with those without a DNAR order.

The discrepancy between our findings and the literature may be explained by differences in patient populations, study designs, and care pathways. In our study, the DNAR order was already in place before the MEU contacted the patient, so the EM physician had no role in defining it. Moreover, we assessed the effect of this factor in relation to initial hospital admission following prehospital home evaluation. In contrast, most previous work has focused on the risk of readmission or the outcomes after hospitalisation or surgery. A prehospital DNAR order may reflect underlying frailty and limited physiological reserve in our setting and may therefore act as a proxy marker of vulnerability; however, DNAR practices vary across systems and this interpretation should be made with caution. In the prehospital triage context, such vulnerability could plausibly increase the risk of hospital admission when an acute or a critical illness arises. To our knowledge, few if any studies have directly examined the relationship between a pre-existing DNAR order and initial admission decisions in prehospital cohorts. Given the lack of information available in the literature, additional studies should be performed to confirm our findings and to explore whether the DNAR status should be incorporated into prehospital risk-stratification tools to enhance clinical decision-making and resource allocation.

It is also important to note that in the Danish context, a DNAR order does not imply that a patient cannot be admitted to hospital or can only receive limited care. A DNAR order only specifies the approach to cardiopulmonary resuscitation in the event of cardiac arrest. Many patients with a pre-existing DNAR order still receive a full diagnostic work-up and active treatment, including hospital admission, when it is clinically indicated. The decision as to whether a patient is a candidate for admission due to advanced comorbidity or frailty are documented separately (e.g., treatment-limitation or ceiling-of-care notes) and are distinct from the DNAR status.

Our model also suggests that compared with community-dwelling older adults, nursing home residents have lower odds of hospital admission 30 days after an MEU contact. Consistently, Kristensen et al. [31] and Krüger et al. [32] found that nursing home residents have a lower or equivalent risk of readmission or hospitalisation compared with the general older adult population. This reduced risk can be explained by the availability of trained healthcare staff at nursing homes who are always available to perform routine monitoring as well as basic acute interventions. These factors ensure that nursing home residents receive timely care and reduce the need for hospital transfers. By contrast, community-dwelling older adults lack continuous monitoring, which may increase the risk of admission following an MEU contact. These observations align with the systematic review by Konetzka et al. [33], who found that higher healthcare staff-to-patient ratio is the strongest factor associated with reduced hospitalisation of nursing home residents. Based on these differences, it may be useful to check on community-dwelling older adults in the period immediately following an MEU contact. This structured ‘enhanced home care’ could involve daily nursing check-ins, remote monitoring of vital signs, and rapid escalation pathways during the first 72–96 hours to mitigate early deterioration and to reduce unplanned admissions. Prospective trials are necessary to evaluate whether this approach could improve safety and decrease 30-day hospitalisation in community-dwelling older adults.

Our study has several strengths. We focused on a novel care model in Denmark, analysed a relatively large patient cohort, and were able to comprehensively gather prehospital clinical variables. Because we excluded terminally ill patients and those directly admitted to hospital, our cohort provides a more accurate reflection of the target population in which prehospital admission risk stratification is clinically relevant. Finally, our work is novel: to our knowledge, few prior studies have specifically examined hospital admission following prehospital emergency physician assessment at home.

We must also acknowledge several limitations of our study. First, the retrospective, single-centre design may limit the generalisability of our findings. Thus, our findings need to be validated in other regions before the identified associations can be generalised or incorporated into clinical tools. Second, we analysed multiple predictors, but we cannot exclude the effect of unmeasured confounding variables. Specifically, our handling of patients who died within 30 days without any hospital admission may have introduced competing-risk bias because death can occur before and preclude admission. Additionally, we excluded visits involving patients on a terminal/palliative trajectory with a comfort-focused plan; therefore, our findings should not be extrapolated to end-of-life populations. Consequently, our estimates should be interpreted as associations rather than causal effects, again highlighting the need for prospective studies. Finally, patients with suspected time-critical cardiac conditions (e.g., acute coronary syndrome or unstable arrhythmias) are typically conveyed directly to hospital in our system and were therefore not represented/eligible in this cohort. Accordingly, our findings may not generalise to patients with primary cardiac presentations.

## Conclusion

In this retrospective cohort of older adults initially managed at home after an MEU assessment, a pre-existing DNAR order and home (vs nursing home) residence were independently associated with 30-day hospital admission. These findings may help inform risk stratification during physician-led home assessments.

## Electronic supplementary material

Below is the link to the electronic supplementary material.

Supplementary material 1

## Data Availability

Due to GDPR and local institutional policies, the dataset cannot be publicly shared. De-identified data are available from the corresponding author on reasonable request and subject to approval by the Region of Southern Denmark.

## Abbreviations

MEU: Mobile emergency unit
DNAR: Do-not-attempt-resuscitation
POCT: Point-of-care testing
POCUS: Point-of-care ultrasound
ED: Emergency departments
EHR: Electronic health record
DVT: Deep vein thrombosis
GCS: Glasgow Coma Scale
OR: Odds ratios
CI: Confidence intervals
EM: Emergency medicine
IQR: Interquartile range
