# Supplementary material for: 30-day hospital admission among older adults initially managed at home by a mobile emergency unit: a retrospective cohort study
Source: BMC Emerg Med. 2026 Mar 11;26:111. doi: 10.1186/s12873-026-01536-5 (PMC13088410; doi:10.1186/s12873-026-01536-5)
Supplement: Supplementary file 1 — Supplementary material 1 [file 12873_2026_1536_MOESM1_ESM.pdf]

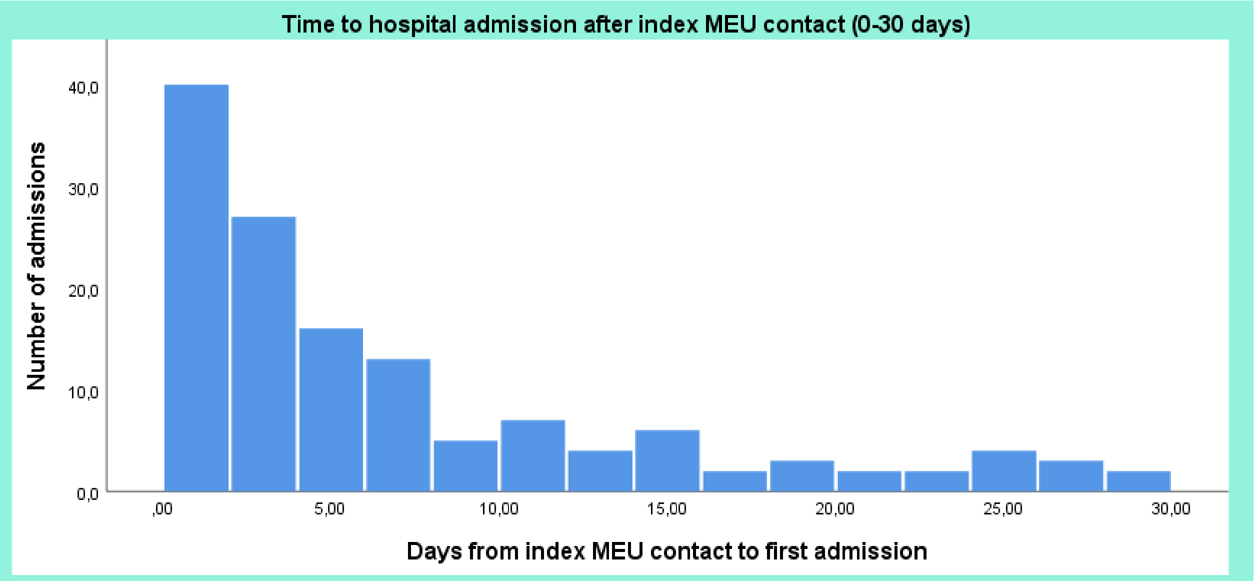

Supplementary Figure S1. Time to hospital admission after the index MEU contact (0–30 days). Bar chart showing the distribution of days from the index MEU contact to the first hospital admission among patients admitted within 30 days. Day 0 indicates same-day admission.
